# Impact of Leaflet-to-Annulus Index on Residual Regurgitation Following Transcatheter Edge-to-Edge Repair of the Tricuspid Valve

**DOI:** 10.3390/jcm13144176

**Published:** 2024-07-17

**Authors:** Fausto Pizzino, Giancarlo Trimarchi, Andreina D’Agostino, Michela Bonanni, Giovanni Benedetti, Umberto Paradossi, Rachele Manzo, Rosangela Capasso, Gianluca Di Bella, Concetta Zito, Scipione Carerj, Sergio Berti, Massimiliano Mariani

**Affiliations:** 1 Fondazione Toscana G. Monasterio, Ospedale del Cuore, 54100 Massa, Italy; fpizzino@ftgm.it (F.P.); adagostino@monasterio.it (A.D.); michelabonanni91@gmail.com (M.B.); giovanni.benedetti@ftgm.it (G.B.); uparadossi@ftgm.it (U.P.); ifcberti@ftgm.it (S.B.); marianims@ftgm.it (M.M.); 2 Department of Clinical and Experimental Medicine, University of Messina, 98100 Messina, Italy; giancarlo.trimarchi18@gmail.com (G.T.); gianluca.dibella@unime.it (G.D.B.); concetta.zito@unime.it (C.Z.); 3 Department of Advanced Biomedical Sciences, Division of Cardiology, Federico II University, 80131 Naples, Italy; rachele4manzo@gmail.com; 4 Department of Clinical and Molecular Medicine, Division of Cardiology, Sapienza University of Rome, 00185 Rome, Italy; capassorosangela@gmail.com

**Keywords:** tricuspid valve, tricuspid regurgitation, transcatheter edge-to-edge repair, leaflet-to-annulus index

## Abstract

**Background**: The mismatch between tricuspid valve (TV) leaflet length and annulus dilation, assessed with the septal–lateral leaflet-to-annulus index (SL-LAI), predicts residual tricuspid regurgitation (TR) following tricuspid transcatheter edge-to-edge-repair (T-TEER). When posterior leaflet grasping is required, the anterior–posterior leaflet-to-annulus index (AP-LAI) may offer additional information. **Methods**: This single-center retrospective cohort study included all patients referred for T-TEER with severe and symptomatic TR with high surgical risk from April 2021 to March 2024. Patients were categorized into ‘optimal result’ (<moderate TR) or ‘suboptimal result’ (≥moderate TR) groups. The SL-LAI and AP-LAI were calculated using pre-procedural transesophageal echocardiography (TEE) measurements. **Results**: Of the 25 patients, 12 had suboptimal post-procedural results, while 13 showed optimal outcomes. The optimal result group showed a higher prevalence of type IIIA-IIIB TV morphology (85% vs. 45%, *p* < 0.05), a wider SL annulus diameter (42.5 ± 5 vs. 37 ± 5 mm, *p* < 0.05), and a longer posterior leaflet length (28 ± 4 vs. 22 ± 5 mm, *p* < 0.01). The SL-LAI was lower in the optimal group (1 ± 0.2 vs. 1.2 ± 0.32, *p* < 0.05), while the AP-LAI was higher (0.7 ± 0.1 vs. 0.5 ± 0.2, *p* < 0.05). ROC curve analysis showed that the AUC for the AP-LAI was 0.769 (95% CI 0.51–0.93, *p* < 0.05) and Youden test identified the best cut-off value <0.5 (sensitivity 50% and specificity 100%) for a suboptimal result. The SL-LAI showed a very low AUC in predicting suboptimal results (0.245, 95% CI 0.08–0.47). Comparing the two ROC curves, we showed that AUC difference is significant with the AP-LAI showing the best association with the outcome (*p* = 0.01)**. Conclusions**: The AP-LAI and SL-LAI can help in predicting post T-TEER results, ameliorating patients’ outcomes and avoiding futile procedures.

## 1. Introduction

High-grade tricuspid regurgitation (TR) is becoming more prevalent due to an aging population with increasing comorbidities, affecting approximately 3% of individuals over 65 [1], and carries a poor prognosis without appropriate treatment [2].

Once considered benign, recent data show that TR is linked to higher morbidity and mortality, even in patients without left ventricular dysfunction [3,4]. Tricuspid valve surgery for isolated TR is rare in the elderly due to high postoperative mortality [5], and medical treatment only manages symptoms. Consequently, many symptomatic patients receive no specific treatment [6]. However, transcatheter edge-to-edge tricuspid valve repair (T-TEER) has recently been recognized as a safe and effective option for inoperable patients [7,8] and is now included in current guidelines [9].

Current T-TEER treatment options include the TriClip (Abbott Vascular, Santa Clara, CA, USA) or the PASCAL systems (Edward Lifesciences, Irvine, CA, USA) widely used for their safety, ease of use, and availability [10,11]. Both methods generally work by bridging the septal leaflet with the anterior leaflet using paddles and clasps. However, in cases of complex valve anatomy, the posterior leaflet may also be grasped, and the implantation of a second or third device might be necessary [12]. Greater TR severity reduction post T-TEER is associated with improved exercise tolerance, symptom relief, survival rates, and clinical outcomes [7,13]. Therefore, identifying anatomical and functional parameters that predict TR reduction following the procedure is critical for enhancing patient outcomes and for better planning the procedure itself, avoiding futility.

Tricuspid annular (TA) dilation is a common morphological feature of functional TR [14,15]. TA dilation occurs secondary to right ventricular and atrial dilation, leading to a reduced coaptation surface area. However, varying degrees of TR can be observed in patients with the same extent of TA dilation, potentially due to differences in TV leaflet size. Larger TV leaflets may theoretically prevent TR development when the TA dilates, whereas relatively smaller TV leaflets compared to the TA dilation can exacerbate TR development [16,17].

This mismatch between tricuspid leaflet length and annulus dilation is an important anatomical feature linked to TR development. It can be assessed using the septal–lateral leaflet-to-annulus index (SL-LAI) as proposed by Tanaka et al., who highlighted its role in predicting significant residual TR after T-TEER [16].

In cases where TV anatomy requires grasping the posterior leaflet, this index may be less useful in predicting residual TR after the procedure. In such contexts, assessing the anterior–posterior leaflet-to-annulus index (AP-LAI) may provide additional information. In this study, we measured both the SL-LAI and the AP-LAI to assess the leaflet-to-annulus mismatch, and we investigated the added value of the latter index in predicting significant residual TR after T-TEER.

## 2. Materials and Methods

The study is designed as a single-center retrospective cohort study including all patients referred to our center with severe or more symptomatic TR despite guideline-directed medical therapy (GDMT), with high surgical risk and judged suitable for TEER suitable for TEER, from April 2021 to March 2024. TEER suitability was explored by experienced physicians in imaging and in TEER intraprocedural guidance. The exclusion criteria were (1) the poor quality of echocardiographic images; (2) the absence of post-procedural echocardiographic evaluation; (3) a coaptation gap greater than 7 mm; and (4) other concomitant valvular diseases with more than moderate severity.

All patients underwent a pre-procedural transesophageal echocardiogram (TEE) performed with a commercial machine equipped with a 3-dimensional (3D) 7-2 Mhz probe (Philips, Amsterdam, The Netherlands). Moreover, patients underwent to comprehensive transthoracic echocardiogram (TTE) performed with the same machine with a phased array 5-1 Mhz probe (Philips, The Netherlands).

TEE was performed from middle esophageal, deep esophageal and transgastric views optimized for right chamber and tricuspid valve studies, according to the American Society of Echocardiography (ASE) guidelines [18]. TR was judged according to ERO, evaluated with the PISA method, and to the VC area evaluated on 3D images with multiplanar reconstruction (MPR). All measures of tricuspid annulus and leaflet length were performed via 3D images with MPR. The identification of the morphology of leaflets and of the localization of jets were performed on both 2-dimensional (2D) and 3D images obtained by TEE. We collected different anatomical and functional echocardiography-derived parameters, aiming to explore their association with the post-procedural result. Among these, we included the following in the study: TR etiology, tricuspid valve morphology, tricuspid annulus diameters, leaflet length, septal–lateral leaflet-to-annulus index (SL-LAI), jet localization, tricuspid annulus plane systolic excursion (TAPSE), fractional area change (FAC), systolic pulmonary artery pressure (SPAP), right ventricle end-diastolic area (RV-EDA), and right atrium area (RA-A). TR etiology was divided only in primary (flail or prolapse) and secondary to atrial dilatation, and no other forms of TR were present in our population. To assess tricuspid valve morphology, we referred to the classification proposed by Hahn et al. [19] which clusters valve morphology according to the number of leaflets and to the localization of the clefted leaflets.

Due to the limited number of patients, we chose to condense the types of tricuspid valve morphology into three categories. The first category includes types I and II, the second, types IIIA and IIIB, and the third, types IIIC and IV, reflecting increasing levels of morphological complexity. Annulus diameters were measured at end-diastole using the QRS reference. Leaflet lengths were measured at the end of systole. In cases of a split leaflet, the length of the most representative portion was considered or the wider of the two when their perimeters were similar.

**Figure 1:**
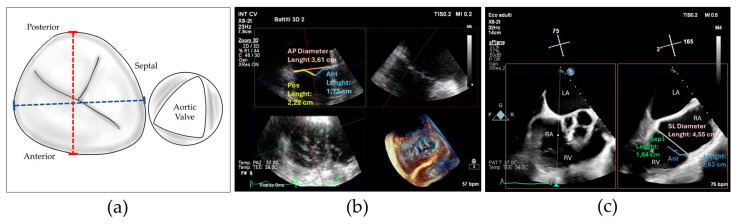
(**a**) Drawing showing tricuspid valve annulus and leaflets and the measures of antero-posterior diameter (red dotted line) and septal–lateral diameter (blue dotted line); (**b**) 3D transesophageal echocardiography with MPR reconstruction showing antero-posterior diameter measure (red) and anterior (green) and posterior (blue) leaflet measurement for computing the antero-posterior leaflet-to-annulus index; (**c**) 2D transesophageal echocardiography with orthogonal cross-section view showing the measure of septal–lateral diameter (red), septal (green) and anterior (blue) leaflet diameter for computing septal–lateral leaflet-to-annulus index; Abbreviations: Ant—anterior, AP—antero-posterior, Pos—posterior, Sep—septal, LA—left atrium, RA—right atrium, RV—right ventricle, SL—septal–lateral.

The SL-LAI was calculated by the sum of the lengths of septal and anterior leaflets normalized for the SL annulus diameter as previously described [16]. However, in our center experience, many patients treated with initial antero-septal grasping showed an intraprocedural persistence of significant TR and needed a second device implantation, often grasping the posterior leaflet. Therefore, we developed a novel modified leaflet-to-annulus index (LAI) derived from the sum of the anterior and posterior leaflets, normalized for the antero-posterior annulus diameter and we defined it as the AP-LAI (see Figure 1).

**Figure 2:**
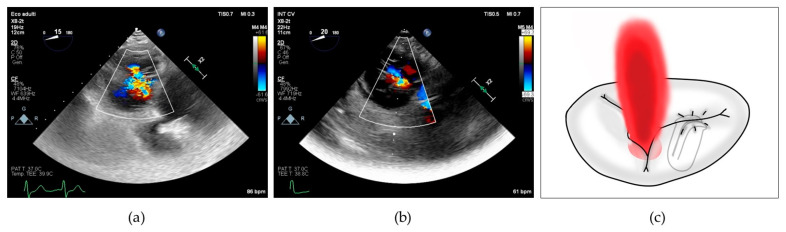
(**a**) 2D transesophageal echocardiographic transgastric view showing complex tricuspid regurgitation; (**b**) 2D transesophageal echocardiographic transgastric view showing central + anterior–septal tricuspid regurgitation; (**c**) drawing showing residual tricuspid regurgitation between anterior and posterior leaflets after implantation of a single clip grasping anterior and septal leaflet.

To better characterize this last concept, we collected the prevalence of posterior grasping needed in our population. Jet localizations were divided in 4 categories: isolated central when the prevalent jet was central, isolated antero-septal (AS) when the prevalent jet was located between anterior and septal leaflets, central + AS when both components coexisted and neither one was prevalent on the other, and finally, we included in a last category named as “complex” all the jet localizations which cannot be included in the others, encompassing regurgitation characterized by multiple jets located in the posterior commissures and star-shaped jets (see Figure 2).

We collected also some essential procedural data including the type of implanted device that in our center can include NT, NTW, XT and XTW Mitraclip^®^ (Abbott Vascular, Santa Clara, CA, USA) and Pascal^®^ P10 or ACE (Edwards Lifesciences, Irvine, CA, USA). Procedures were performed under general anesthesia with 3D TEE and fluoroscopic guidance. The number of implanted devices was reported as well as total fluoroscopy time length.

Implantation success was defined as the successful delivery and deployment of one or more clips to achieve leaflet approximation and retrieval of the delivery system. Post-procedural success was defined as at least one-grade reduction in TR severity upon discharge. An ‘optimal result’ is achieved if the residual TR degree is less than moderate, while a ‘suboptimal result’ is indicated if the residual TR degree is moderate or greater.

### Statistical Analysis

Continuous variables were expressed as means ± SD or median (25th; 75th percentiles) depending on normality. Categorical variables are expressed as numbers and percentages n (%). Normal distribution was assessed using the Shapiro–Wilk test. The comparison between continuous variables was performed by Student’s independent samples *t*-test or Wilcoxon test according to distribution. The association of the variables with the outcome was performed with receiving operating characteristic (ROC) curves and the area under the curve (AUC), and the graphical representation of the curves was calculated by the method of DeLong. The Youden test was used to identify the best cut-off value according to sensitivity and specificity. A comparison of the AUC of the ROC curves was used to measure differences between the variables. All tests were performed as two-sided. Statistical significance was considered for a *p* value < 0.05. The statistical software applications used were SPSS version 23 (IBM Corp. 2015. Armonk, NY, USA, 2015), STATA/MP 13.0 (College Station, TX, USA) and MedCalc version 14.8.1 (MedCalc Software bvba, Ostend, Belgium; 2014).

## 3. Results

Twenty-five patients were enrolled in our center to undergo TEER for symptomatic ≥ severe TR. Baseline characteristics of the patients are reported in Table 1. The mean age was 71 ± 6 years, with 40% being male. All patients were symptomatic despite receiving guideline-directed medical therapy (GDMT) and were classified as NYHA class II (48%) or III (52%).

The echocardiographic parameters are listed in Table 2. Regarding TR etiology, 92% of patients showed atriogenic tricuspid regurgitation as the main mechanism of regurgitation and showed type IIIA-IIIB morphology (67%). It is worth noting that our population was characterized by complex jet localization, with most patients having more than one jet and at least one jet involving posterior commissures. Functional RV parameters were in the normal range (TAPSE and FAC), while RA presented significant dilatation. Interestingly, tricuspid annulus was not particularly dilated, with a mean SL diameter of 39 ± 5 mm and AP diameter of 41 ± 7 mm. The SL-LAI and AP-LAI were calculated with values of AP-LAI lower than the SL-LAI (0.6 ± 0.2 vs. 1.1 ± 0.2).

During the procedure, all patients underwent a successful grasping of leaflets and 60% of them received two devices, with only 12% receiving three devices and 28% receiving only one device. Interestingly, 60% of our population presented an intraprocedural need of posterior leaflet grasping, usually with the second implanted device.

After the procedure, 23 patients had a reduction in the degree of TR: 13 patients (52%) showed a reduction to less than moderate, while 12 patients had a residual degree of TR more than or equal to moderate; the two groups were respectively defined as “optimal result” and “suboptimal result”.

Clustering the population according to the post-TEER result, we revealed that no significant differences were identifiable among the clinical features.

Among anatomical and functional features regarding tricuspid valve and annulus, we observed a higher prevalence of patients with more complex morphological classes of the tricuspid valve in the optimal result group (type IIIA-IIIB 85% vs. 45%, *p* < 0.05); otherwise, patients with a suboptimal result had a higher prevalence of type I-II tricuspid morphology (8% vs. 54%). Pre-procedural TR severity was not different between the groups, suggesting the absence of influence on the post-procedural result. Interestingly, the SL annulus diameter and posterior leaflet were wider in patients with a post-TEER optimal result (42.5± vs. 37 ± 5, *p* < 0.05 and 28 ± 4 mm vs. 22 ± 5 mm, *p* < 0.01, respectively). In contrast, the AP annulus diameter was higher in the suboptimal group even though this difference did not reach statical significance (42 ± 8 mm vs. 40 ± 5 mm).

**Figure 3:**
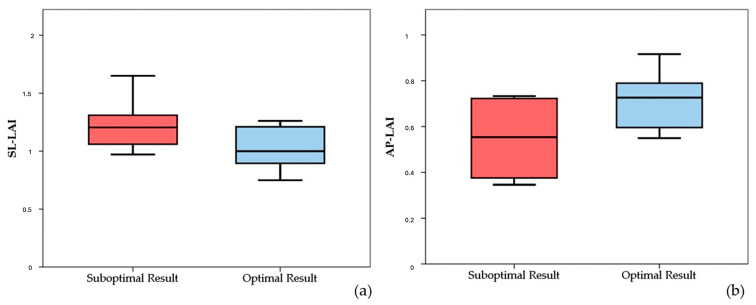
Boxplots showing differences in SL-LAI (**a**) and AP-LAI (**b**) in the two groups of patients with suboptimal and optimal results. SL-LAI—septal–lateral leaflet-to-annulus index, AP-LAI—antero-posterior leaflet-to-annulus index.

It is worth noting that both the SL-LAI and AP-LAI were significantly different in the two groups; however, the group with suboptimal results had a higher SL-LAI, an unexpected result since this condition should theoretically represent a more favorable anatomy for TEER according to the previous literature. Conversely, AP-LAI was significantly lower in the suboptimal result group (0.5 ± 0.2 vs. 0.7 ± 0.1, *p* < 0.05) (see Figure 3).

Jet location did not show difference in the distribution among the two groups, even though patients with complex jets showed a trend to be more prevalent in the suboptimal result group (64% vs. 46%). For details, refer to Table 2.

Among the other echocardiographic features of right chambers, TAPSE was significantly reduced (18 ± 4 mm vs. 23 ± 3 mm, *p* < 0.01) and FAC tended to be lower in patients with a suboptimal result, while other measures including FAC, SPAP, TAPSE/SPAP ratio, RV-ED area and RA area were not different, as shown in Table 2.

Regarding procedural features, fluoroscopy time was longer in the suboptimal group (36 (24–51) minu) compared to the optimal group (26 (19–39) min), likely due to more complex and challenging procedures, although this difference was not statistically significant. The number and types of implanted devices were not different among the groups. Interestingly, we observe a trend of more prevalent posterior grasping in the suboptimal result group in comparison to the optimal result group (75% vs. 46%), as shown in Table 3.

**Table 3:** Procedural features of patients who underwent tricuspid transcatheter edge-to-edge repair, distinguishing those with optimal post-procedural result from those with suboptimal optimal post-procedural result.

Procedural Features	Whole Population (n = 25)	Suboptimal Result (n = 12)	Optimal Result (n = 13)	*p*
PASCAL ACE	15 (60%)	7 (58%)	8 (61%)	0.9
XTW	9 (36%)	5 (42%)	4 (31%)	0.9
PASCALE ACE + P10	1 (4%)	0	1 (8%)	1
Posterior grasping	15 (60%)	9 (75%)	6 (46%)	0.2
Number of implanted devices				
1	7 (28%)	3 (25%)	4 (31%)	0.9
2	15 (60%)	8 (67%)	7 (54%)	0.9
3	3 (12%)	1 (8%)	2 (15%)	0.6
Fluoroscopy duration (min)	26 (19–39)	36 (24–51)	24 (12–28)	0.6

**Figure 4:**
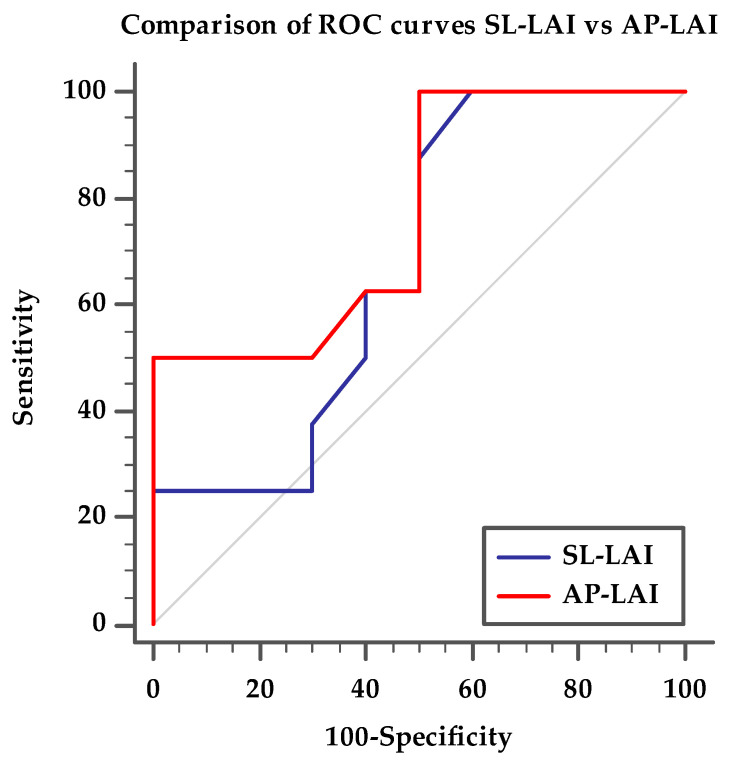
Comparison of ROC curves for AP-LAI and SL-LAI, discriminating patients with optimal post-procedural result from patients with suboptimal post-procedural results. ROC curve for SL-LAI with AUC 0.675 (95% CI 0.42–0.87), ROC curve for AP-LAI with AUC 0.769 (95% CI 0.51–0.93, *p* < 0.05), *p* < 0.05 for ROC curve pairwise comparison.

ROC curve analysis showed that AUC for AP-LAI was 0.769 (95% CI 0.51–0.93, *p* < 0.05) and Youden test identified the best cut-off value < 0.5 (sensitivity 50%, specificity 100%) for a suboptimal result. SL-LAI showed lower AUC in predicting suboptimal result (0.675, 95% CI 0.42–0.872). Comparing the two ROC curves, we showed that AUC difference is significant, with AP-LAI showing the best association to the outcome (*p* < 0.05), as detailed in Figure 4.

## 4. Discussion

The main finding of our study indicates that in patients undergoing T-TEER, the use of AP-LAI is a better predictor of the procedure’s outcome compared to SL-LAI. Specifically, the finding of an AP-LAI < 0.5 has 100% specificity in predicting a suboptimal result with 50% sensitivity. The elevated specificity provides our finding with an elevated negative predictive value; therefore, finding a value > 0.5 theoretically excludes a suboptimal result after procedure.

Selecting suitable patients for tricuspid TEER is crucial for optimal TR reduction and several studies have demonstrated that residual TR with a degree of moderate or greater severity adversely affects clinical outcomes [7,20]. Given the increasing demand for tricuspid interventions, identifying predictors of substantial TR reduction after the procedure is essential [15].

To prevent ineffective procedures, numerous echocardiographic exclusion criteria have been established based on key studies like the CLASP TR easy feasibility study [11] and the TRILUMINATE Pivotal Trial [7,21,22]. These criteria aim to identify patients who are unlikely to benefit from the procedure. For example, the CLASP TR study excludes patients with severe left ventricular dysfunction where the left ventricular ejection fraction (LVEF) is below 30%, severe right ventricular dysfunction, coaptation gaps greater than 10 mm, and leaflet lengths shorter than 8 mm. The TRILUMINATE trial sets even stricter criteria, excluding patients with severe LVEF below 20% and coaptation gaps over 20 mm [7]. Both trials share common exclusion factors, including those requiring a correction for left-sided or pulmonary valve issues, a history of previous tricuspid valve procedures, tricuspid stenosis, rheumatic leaflet degeneration, moderate to severe calcification in critical areas, an Ebstein anomaly, pulmonary artery systolic pressure over 70 mmHg, and cardiac implanted electronic devices (CIEDs) that could impede proper device placement.

Despite following these recommendations, a suboptimal procedural outcome is observed in a significant percentage of patients undergoing tricuspid TEER, with residual moderate or greater tricuspid regurgitation occurring in about 30–50% of cases [23,24].

In this context, evaluating the mismatch between leaflet length and annulus dilation may aid in better patient selection and prevent futile interventions. This assessment is supported by Tanaka et al., who demonstrated that a lower LAI is associated with moderate or greater residual TR after TEER, regardless of the baseline TR grade and other anatomical parameters [16].

To justify this result, the authors provided several possible explanations. Firstly, despite annulus dilatation, an excess of leaflet tissue (higher LAI) can facilitate coaptation after edge-to-edge repair. Conversely, a short leaflet length (lower LAI) may impede successful clip insertion, forcing clinicians to place devices farther from the main TR jet, resulting in an ineffective reduction in TR. Furthermore, advanced TA dilation can broaden the TR jet area, extending it from the center or anteroseptal commissure to the anteroposterior or posterior–septal commissures of the TV. This expanded TR location has been identified as a predictor of procedural failure [23].

In their study, Tanaka et al. used the SL-LAI to predict the severity of post-procedural TR regurgitation and found that higher SL-LAI values correlated with better procedural outcomes [16]. Contrarily, our study showed that patients with optimal procedural results had lower SL-LAI values but higher AL-LAI values.

This discrepancy could be attributed to the fact that in our sample, patients with optimal results had a significantly larger baseline SL annulus diameter and a similar, though slightly lower, AP annulus diameter compared to those with suboptimal results. Furthermore, patients with optimal procedural outcomes had a longer posterior leaflet, suggesting more effective grasping. In our population, 60% of patients underwent posterior leaflet grasping, whereas only 20% did in the population studied by Tanaka [16]. The need to grasp the posterior leaflet in our population indicates a more complex and challenging anatomy, as shown by 67% of patients having type IIIA-IIIB tricuspid anatomy according to Hahn et al.’s classification [19]. This is further supported by the requirement of more than one device in 72% of cases. In this context, evaluating the AP-LAI alongside the SL-LAI can provide valuable insights for procedural planning and optimal patient selection. While most clip devices were implanted along the anteroseptal coaptation line [25], occasionally, to improve outcomes, the posterior leaflet, especially at the anteroposterior commissure, was grasped when feasible.

**Figure 5:**
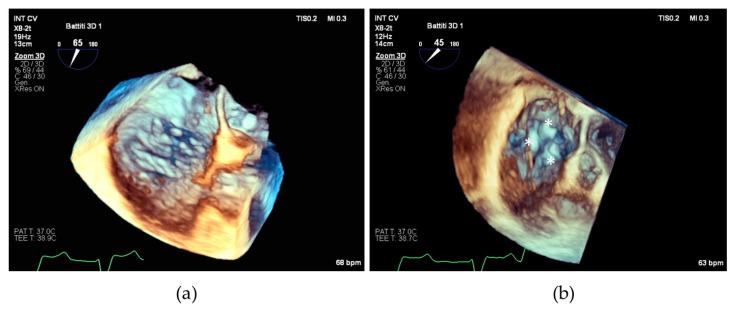
(**a**) Transesophageal echocardiographic in the 3D zoom modality showing complex tricuspid valve morphology; (**b**) the same valve after repair with implantation of three devices individuated by asterisks.

We believe that our results can be particularly useful in planning the interventional procedure, especially in patients with complex tricuspid valve anatomies, who may require posterior grasping to optimize procedural outcomes (see Figure 5).

The results of our research can assist healthcare professionals in choosing the most suitable devices for tricuspid TEER intervention in clinical settings in order to maximize TR reduction. In recent times, a number of innovative transcatheter devices specifically designed for TR treatment have been developed [26]. One notable advancement is the introduction of new edge-to-edge repair devices which have the capability to independently grasp the leaflets of the tricuspid valve, thereby improving the accuracy of a clip insertion [26]. This unique feature is particularly beneficial when there is a need to grasp the posterior leaflet as it can result in a more effective reduction in TR. Furthermore, these devices are also effective in cases with large coaptation gaps and significant tethering, further enhancing their utility in clinical practice.

## 5. Conclusions

In conclusion, assessing leaflet length-to-annulus mismatch through the SL-LAI and AP-LAI is highly valuable for planning interventional procedures and predicting post-TEER residual TR. An AP-LAI evaluation is particularly beneficial for patients with complex tricuspid valve anatomies, where posterior grasping is often required to optimize procedural outcomes. Our findings demonstrate that AP-LAI, in particular, could aid in enhancing procedural planning and predicting post-intervention results. By offering a more precise method for device selection, our research supports healthcare professionals in choosing the most appropriate devices for tricuspid TEER interventions, ultimately aiming to achieve maximal TR reduction and improve patient outcomes in clinical settings.

## 6. Study Limitations

Several limitations to this study should be acknowledged. First, it was a single-center, retrospective study with a relatively small number of participants. The study’s statistical power was limited by the small number of participants. Consequently, advanced statistical analyses to independently assess the variables’ association with the outcome were not feasible, possibly biasing the results. To address this issue, multi-center studies on larger cohorts are recommended. Additionally, it is important to consider that the valvular anatomies and locations of regurgitation jets in our patients undergoing T-TEER were highly complex. This complexity mandated the use of more than one device in 72% of cases. For 60% of these cases, posterior leaflet grasping was also necessary, in addition to the standard anterior and septal leaflet grasping, which is more conventionally executed. These factors could have influenced the outcomes and may limit the generalizability of the findings. The study results are limited to a parametric echo-derived post-procedural result. No clinical outcomes were collected in our study and so we could not describe the eventual clinical benefit derived from a successful procedure. Further studies with larger, multi-center cohorts are warranted to validate these results.

## Figures and Tables

**Table 1:** Clinical features of patients who underwent tricuspid transcatheter edge-to-edge repair, distinguishing those with optimal post-procedural result from those with suboptimal optimal post-procedural result.

Clinical Features	Whole Population (n = 25)	Suboptimal Result (n = 12)	Optimal Result (n = 13)	*p*
Age (y)	71 ± 6	81 ± 5	77 ± 7	0.1
BSA (m^2^)	1.78 ± 0.2	1.8 ± 0.2	1.8 ± 0.2	0.6
Gender (M)	10 (40%)	4 (33%)	6 (46%)	0.4
Hypertension	16 (64%)	9 (75%)	7 (54%)	0.2
Dyslipidemia	10 (40)	5 (42%)	5 (38%)	0.6
Diabetes	1 (4%)	0	1 8(%)	0.5
NYHA II	12 (48%)	6 (50%)	6 (46%)	0.9
NYHA III	13 (52%)	6 (50%)	7 (53%)	0.9
MRAs	12 (48%)	6 (50%)	6 (46%)	0.6
ACEi-ARBs-ARNI	11 (44%)	6 (50%)	5 (38%)	0.4
SGLT2i	5 (20%)	4 (33%)	1 (8%)	0.1
BB	18 (72%)	10 (83%)	8 (61%)	0.2

Abbreviations: BSA—body surface area, NYHA—New York Heart Association, MRA—mineralocorticoid receptor antagonist, ACEi—angiotensin-converting enzyme inhibitor, ARBs—angiotensin receptor blockers, ARNI—angiotensin receptor–neprilysin inhibitor, SGLT2i—sodium–glucose cotransporter 2 inhibitor, BB—beta adrenergic blocker.

**Table 2:** Echocardiographic features of patients who underwent tricuspid transcatheter edge-to-edge repair, distinguishing those with optimal post-procedural result from those with suboptimal optimal post-procedural result.

Echocardiographic Features	Whole Population (n = 25)	Suboptimal Result (n = 12)	Optimal Result (n = 13)	*p*
Primary Etiology of TR	2 (8%)	0	2 (15%)	0.9
Atriogenic Etiology of TR	23 (92%)	12(100%)	11 (85%)	0.5
Type I-II	7 (29%)	6 (50%)	1 (8%)	<0.05
Type IIIA-IIIB	17 (71%)	6 (50%)	11 (85%)	<0.05
Type IIIc-IV	1 (4%)	0	1 (8%)	1
AP Annulus (mm)	41 ± 7	42 ± 8	40 ± 5	0.5
SL Annulus (mm)	39 ± 5	37 ± 5	42 ± 5	<0.05
Anterior Leaflet Length (mm)	24 ± 4	24 ± 4	24 ± 5	0.8
Septal Leaflet Length (mm)	19 ± 3	20 ± 4	18 ± 3	0.2
Posterior Leaflet Length (mm)	26 ± 5	22 ± 5	28 ± 4	<0.01
SL-LAI	1.1 ± 0.2	1.2 ± 0.32	1 ± 0.2	<0.05
AP-LAI	0.6 ± 0.2	0.5 ± 0.2	0.7 ± 0.1	<0.05
Jet Location				
Isolated Central Jet	4 (16%)	1 (8%)	3 (23%)	0.4
Isolated AS Jet	2 (8%)	1 (8%)	1 (8%)	1
Central + AS Jet	7 (28%)	3 (24%)	4 (31%)	0.8
Complex Jet Location	13 (52%)	7 (64%)	6 (46%)	0.4
Jet Severity				
Severe	14 (56%)	6 (50%)	8 (61%)	
Massive	7 (28%)	5 (41%)	2 (16%)	0.3
Torrential	4 (16%)	1 (8%)	3 (12%)	
EROA (cm^2^)	0.6 (0.57–0.8)	0.6 (0.58–0.78)	0.65 (0.52–0.97)	0.5
Right Chambers’ Features				
TAPSE (mm)	20 ± 4	18 ± 4	23 ± 3	<0.01
FAC (%)	41 ± 12	37 ± 12	44 ± 12	0.2
SPAP (mmHg)	42 ± 10	40 ± 12	44 ± 8	0.4
TAPSE/SPAP	0.5 ± 0.1	0.5 ± 0.1	0.5 ± 0.1	0.3
RV-EDA (cm^2^)	22 ± 5	22 ± 5	22 ± 5	0.8
RA-A (cm^2^)	33 ± 7	32 ± 5	35 ± 9	0.2

Abbreviations: TR—tricuspid classification, AP—antero-posterior, SL—septal–lateral, LAI—leaflet-to-annulus index, AS—antero-septal, EROA—effective regurgitant orifice area, TAPSE—tricuspid annular plane excursion, FAC—fractional annular plane, SPAP—systolic pulmonary artery pressure, RV-EDA—right ventricle end-diastolic area, RA-A—right atrium area.

## Data Availability

Data are available on a reasonable request to the authors.

## References

[1] Topilsky Y., Maltais S., Medina Inojosa J., Oguz D., Michelena H., Maalouf J., Mahoney D.W., Enriquez-Sarano M. (2019). Burden of Tricuspid Regurgitation in Patients Diagnosed in the Community Setting. JACC Cardiovasc. Imaging.

[2] Nishiura N., Kitai T., Okada T., Sano M., Miyawaki N., Kim K., Murai R., Toyota T., Sasaki Y., Ehara N. (2023). Long-Term Clinical Outcomes in Patients with Severe Tricuspid Regurgitation. J. Am. Heart Assoc..

[3] Topilsky Y., Inojosa J.M., Benfari G., Vaturi O., Maltais S., Michelena H., Mankad S., Enriquez-Sarano M. (2018). Clinical Presentation and Outcome of Tricuspid Regurgitation in Patients with Systolic Dysfunction. Eur. Heart J..

[4] Wang N., Fulcher J., Abeysuriya N., McGrady M., Wilcox I., Celermajer D., Lal S. (2019). Tricuspid Regurgitation Is Associated with Increased Mortality Independent of Pulmonary Pressures and Right Heart Failure: A Systematic Review and Meta-Analysis. Eur. Heart J..

[5] Kawsara A., Alqahtani F., Nkomo V.T., Eleid M.F., Pislaru S.V., Rihal C.S., Nishimura R.A., Schaff H.V., Crestanello J.A., Alkhouli M. (2021). Determinants of Morbidity and Mortality Associated with Isolated Tricuspid Valve Surgery. J. Am. Heart Assoc..

[6] Enriquez-Sarano M., Messika-Zeitoun D., Topilsky Y., Tribouilloy C., Benfari G., Michelena H. (2019). Tricuspid Regurgitation Is a Public Health Crisis. Prog. Cardiovasc. Dis..

[7] Sorajja P., Whisenant B., Hamid N., Naik H., Makkar R., Tadros P., Price M.J., Singh G., Fam N., Kar S. (2023). Transcatheter Repair for Patients with Tricuspid Regurgitation. N. Engl. J. Med..

[8] Lurz P., Besler C., Schmitz T., Bekeredjian R., Nickenig G., Möllmann H., von Bardeleben R.S., Schmeisser A., Atmowihardjo I., Estevez-Loureiro R. (2023). Short-Term Outcomes of Tricuspid Edge-to-Edge Repair in Clinical Practice. J. Am. Coll. Cardiol..

[9] Vahanian A., Beyersdorf F., Praz F., Milojevic M., Baldus S., Bauersachs J., Capodanno D., Conradi L., De Bonis M., De Paulis R. (2022). 2021 ESC/EACTS Guidelines for the Management of Valvular Heart Disease. Eur. Heart J..

[10] Prandi F.R., Lerakis S., Belli M., Illuminato F., Margonato D., Barone L., Muscoli S., Chiocchi M., Laudazi M., Marchei M. (2023). Advances in Imaging for Tricuspid Transcatheter Edge-to-Edge Repair: Lessons Learned and Future Perspectives. J. Clin. Med..

[11] Kodali S., Hahn R.T., Eleid M.F., Kipperman R., Smith R., Lim D.S., Gray W.A., Narang A., Pislaru S.V., Koulogiannis K. (2021). Feasibility Study of the Transcatheter Valve Repair System for Severe Tricuspid Regurgitation. J. Am. Coll. Cardiol..

[12] Agricola E., Asmarats L., Maisano F., Cavalcante J.L., Liu S., Milla F., Meduri C., Rodés-Cabau J., Vannan M., Pibarot P. (2021). Imaging for Tricuspid Valve Repair and Replacement. JACC Cardiovasc. Imaging.

[13] Kodali S.K., Hahn R.T., Davidson C.J., Narang A., Greenbaum A., Gleason P., Kapadia S., Miyasaka R., Zahr F., Chadderdon S. (2023). 1-Year Outcomes of Transcatheter Tricuspid Valve Repair. J. Am. Coll. Cardiol..

[14] Badano L.P., Hahn R., Rodríguez-Zanella H., Araiza Garaygordobil D., Ochoa-Jimenez R.C., Muraru D. (2019). Morphological Assessment of the Tricuspid Apparatus and Grading Regurgitation Severity in Patients with Functional Tricuspid Regurgitation: Thinking Outside the Box. JACC Cardiovasc. Imaging.

[15] Taramasso M., Gavazzoni M., Pozzoli A., Dreyfus G.D., Bolling S.F., George I., Kapos I., Tanner F.C., Zuber M., Maisano F. (2019). Tricuspid Regurgitation: Predicting the Need for Intervention, Procedural Success, and Recurrence of Disease. JACC Cardiovasc. Imaging.

[16] Tanaka T., Sugiura A., Kavsur R., Vogelhuber J., Öztürk C., Becher M.U., Zimmer S., Nickenig G., Weber M. (2022). Leaflet-to-Annulus Index and Residual Tricuspid Regurgitation Following Tricuspid Transcatheter Edge-to-Edge Repair. EuroIntervention.

[17] Afilalo J., Grapsa J., Nihoyannopoulos P., Beaudoin J., Gibbs J.S.R., Channick R.N., Langleben D., Rudski L.G., Hua L., Handschumacher M.D. (2015). Leaflet Area as a Determinant of Tricuspid Regurgitation Severity in Patients with Pulmonary Hypertension. Circ. Cardiovasc. Imaging.

[18] Hahn R.T., Abraham T., Adams M.S., Bruce C.J., Glas K.E., Lang R.M., Reeves S.T., Shanewise J.S., Siu S.C., Stewart W. (2013). Guidelines for Performing a Comprehensive Transesophageal Echocardiographic Examination: Recommendations from the American Society of Echocardiography and the Society of Cardiovascular Anesthesiologists. J. Am. Soc. Echocardiogr..

[19] Hahn R.T., Weckbach L.T., Noack T., Hamid N., Kitamura M., Bae R., Lurz P., Kodali S.K., Sorajja P., Hausleiter J. (2021). Proposal for a Standard Echocardiographic Tricuspid Valve Nomenclature. JACC Cardiovasc. Imaging.

[20] Dimitriadis K., Pyrpyris N., Aznaouridis K., Iliakis P., Valatsou A., Tsioufis P., Beneki E., Mantzouranis E., Aggeli K., Tsiamis E. (2023). Transcatheter Tricuspid Valve Interventions: A Triumph for Transcatheter Procedures?. Life.

[21] Lurz P., Stephan von Bardeleben R., Weber M., Sitges M., Sorajja P., Hausleiter J., Denti P., Trochu J.-N., Nabauer M., Tang G.H.L. (2021). Transcatheter Edge-to-Edge Repair for Treatment of Tricuspid Regurgitation. J. Am. Coll. Cardiol..

[22] Nickenig G., Weber M., Lurz P., von Bardeleben R.S., Sitges M., Sorajja P., Hausleiter J., Denti P., Trochu J.-N., Näbauer M. (2019). Transcatheter Edge-to-Edge Repair for Reduction of Tricuspid Regurgitation: 6-Month Outcomes of the TRILUMINATE Single-Arm Study. Lancet.

[23] Mazzola M., Giannini C., Sticchi A., Spontoni P., Pugliese N.R., Gargani L., De Carlo M. (2024). Transthoracic and Transoesophageal Echocardiography for Tricuspid Transcatheter Edge-to-Edge Repair: A Step-by-Step Protocol. Eur. Heart J. Imaging Methods Pract..

[24] Cepas-Guillen P.L., de la Fuente Mancera J.C., Guzman Bofarull J., Farrero M., Regueiro A., Brugaletta S., Ibañez C., Sanchis L., Sitges M., Sabate M. (2021). Initial Results after the Implementation of an Edge-To-Edge Transcatheter Tricuspid Valve Repair Program. J. Clin. Med..

[25] Taramasso M., Hahn R.T., Alessandrini H., Latib A., Attinger-Toller A., Braun D., Brochet E., Connelly K.A., Denti P., Deuschl F. (2017). The International Multicenter TriValve Registry: Which Patients Are Undergoing Transcatheter Tricuspid Repair?. JACC Cardiovasc. Interv..

[26] Muntané-Carol G., Alperi A., Faroux L., Bédard E., Philippon F., Rodés-Cabau J. (2020). Transcatheter Tricuspid Valve Intervention: Coaptation Devices. Front. Cardiovasc. Med..

